# Improvement Strategies for Stability and Efficiency of Perovskite Solar Cells

**DOI:** 10.3390/nano12193295

**Published:** 2022-09-22

**Authors:** Hongliang Liu, Ling Xiang, Peng Gao, Dan Wang, Jirui Yang, Xinman Chen, Shuti Li, Yanli Shi, Fangliang Gao, Yong Zhang

**Affiliations:** 1 Guangdong Engineering Research Center of Optoelectronic Functional Materials and Devices, South China Normal University, Guangzhou 510631, China; 2 Tianjin Institute of Power Sources, Tianjin 300384, China; 3 Library of South China Agricultural University, Guangzhou 510642, China

**Keywords:** perovskite solar cells, tandem solar cell, solar cell structure, precursor doping

## Abstract

Recently, perovskites have garnered great attention owing to their outstanding characteristics, such as tunable bandgap, rapid absorption reaction, low cost and solution-based processing, leading to the development of high-quality and low-cost photovoltaic devices. However, the key challenges, such as stability, large-area processing, and toxicity, hinder the commercialization of perovskite solar cells (PSCs). In recent years, several studies have been carried out to overcome these issues and realize the commercialization of PSCs. Herein, the stability and photovoltaic efficiency improvement strategies of perovskite solar cells are briefly summarized from several directions, such as precursor doping, selection of hole/electron transport layer, tandem solar cell structure, and graphene-based PSCs. According to reference and analysis, we present our perspective on the future research directions and challenges of PSCs.

## 1. Introduction

Traditional fossil energy has to be replaced by renewable energy sources on an urgent basis due to the limited reserves and environmental pollution. Solar power has garnered significant research attention because of its clean nature, reproducibility and adequacy on our planet [1]. Since Chapin and Pierson developed the first practical single-crystal silicon solar cells and demonstrated the practical utilization of photovoltaic technology that converts solar energy into electricity in 1954, the development of solar cell technology has been extremely rapid [2]. At present, there are three generations of solar cells with different structures, ranging from monocrystalline silicon to nanostructured thin-film, hybrid and organic cells [3]. The first generation mainly consists of monocrystalline and polycrystalline silicon-based solar cells. The second generation is composed of a series of thin-film solar cells, such as *α*-Si, CdTe, GaAs, CIGS and CuGaSe [4,5]. The third-generation includes single-cell or tandem devices based on Cu_2_ZnSnS_4_ (CZTs), Cu_2_ZnSnSe_4_ (CZTSe), quantum dot solar cells, organic dye-sensitized solar cells and perovskite solar cells [6]. Si-based solar cells have occupied the current market due to the solid foundation of long-term development [2]. At present, the maximum efficiency of Si-based solar cells reached 31.25% in 2022. However, any significant advancement has not been achieved due to the material limitations and Shockley-Queisser (S-Q) limits [7].

The perovskite structure has attracted worldwide attention due to its advantages, such as adjustable bandgap, rapid spectral response and solution-based processing. Since the report on initial efficiency of 3.8%, a series of pivotal problems, such as the dissolution of MAPbI_3_ in liquid electrolytes and application of crucial materials (MAPbI_3_ and spiro-MeOTAD), perovskite solar cells have seen an exciting era of rapid development [8]. The efficiency of perovskite-based single-cell increased from 3.8% in 2009 to 25.7% in 2022. Such a rapid increase in conversion efficiency is conducive to the replacement of Si-based solar cells with perovskite solar cells [9].

**Figure 1:**
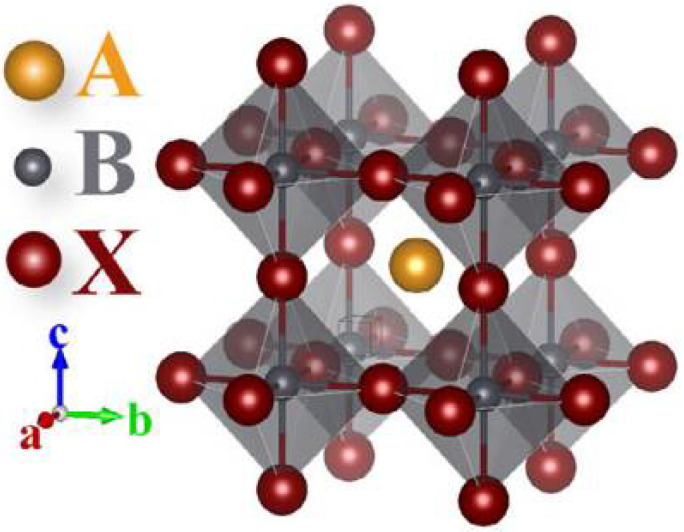
The structure of a typical perovskite crystal: the A ion is a monovalent cation, the B site is mainly composed of some divalent metal cations, and the X position is mainly composed of halide ions of halogen elements [12].

Halide perovskites usually possess a 3D structure [10], as shown in Figure 1, with a chemical composition of ABX_3_, where A represents large-sized cations (CH_3_NH_3_ (MA^+^), formamidinium (FA^+^) and cesium (Cs^+^)), B refers to the divalent metallic cation and X denotes the halide anion (Cl, Br, or I) [11].

Despite the rocketed improvement in power conversion efficiency (PCE) of perovskite solar cells, the instability of the perovskite structure impedes the commercialization of perovskite solar cells (PSCs), which is mostly caused by the presence of erratic organic cations and degradation due to water, light and oxygen [13]. To solve these instability problems, the researchers have adopted different strategies, such as doping and interface engineering [14]. Meanwhile, the hysteresis of J-V curves can be attributed to charge carrier accumulation in the depletion layer or neutral region of the junction, the existence or formation of defect states, and ionic migration, leading to numerous defects, such as inaccurate I-V measurements and wrongly estimated efficiency values [15]. These phenomena have been observed in different photovoltaic architectures and perovskite solar cells.

**Figure 2:**
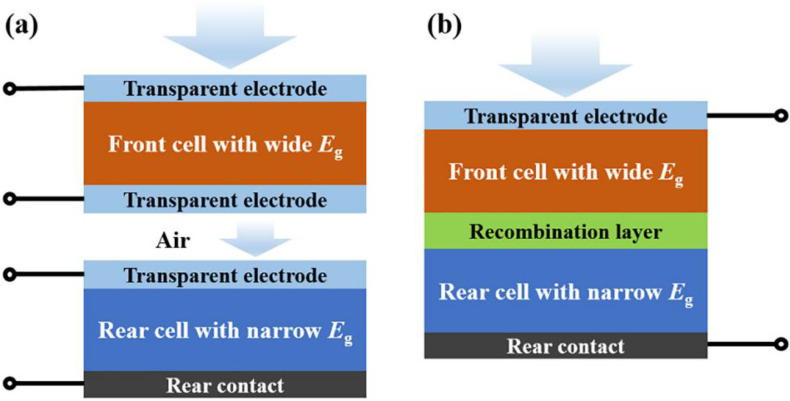
(**a**) Schematic of tandem solar cell with four-port structure, (**b**) Schematic of tandem solar cell with two-port structure [21].

Although the efficiency of a single solar cell continues to increase, it is still difficult to achieve further breakthroughs due to the S-Q limit [16]. At present, tandem cells, where single cells are connected in series, are utilized to break the S-Q limit [17]. The basic principle of a tandem solar cell is shown in Figure 2. It uses sub-cell materials with different bandgaps to absorb light in different wavelength ranges, thereby increasing the absorption range of the spectrum and effectively increasing the open-circuit voltage [18]. Owing to the adjustable bandgap, perovskite is an ideal material for making top or bottom cells of the tandem solar cells. The bandgap can be adjusted by doping different elements. For example, increasing the proportion of Br in ABBr_3_ can increase the bandgap, and increasing the proportion of Sn at the B-site can reduce the bandgap by about 1.18–2.3 eV [19]. There are two basic structures of perovskite cells, i.e., two-terminal (2T) stacked solar cell and four-terminal (4T) stacked solar cell [20].

Correspondingly, the hole transport layers and electron transport layers also play an important role in determining the device efficiency, which is influenced by the charge extraction and charge transportation characteristics [22].

In addition to the PCE and toxicity, large-scale fabrication and stability should also be considered in terms of commercialization [23]. To improve the practicality of PSCs, a vast research effort has been carried out, resulting in a large number of research articles and patents. In this review, we collect and present recent research findings, mainly about the halide PSCs, tandem structure hole transport and electron transport layers.

## 2. Optimize Bandgap and Improve Crystal Quality by Doping Modification

The high-quality perovskite crystal and high-performance electron/hole transport layer (ETL/HTL) play a critical role in determining the photovoltaic device performance. Additive engineering is an effective method to improve the quality of perovskite, ETL and HTL by doping the desired elements or organic ingredients, altering the band structure [24]. There are two main applications for additive engineering strategy. First, the device efficiency is ameliorated, which is related to the optical and electrical properties. Second, the device stability is improved by impeding the degradation of perovskite, which is also termed passivation [25]. Moreover, optimizing bandgap and band alignment by replacing different elements in perovskite is another effective strategy to attain higher PCE.

### 2.1. Bandgap Regulation

**Figure 3:**
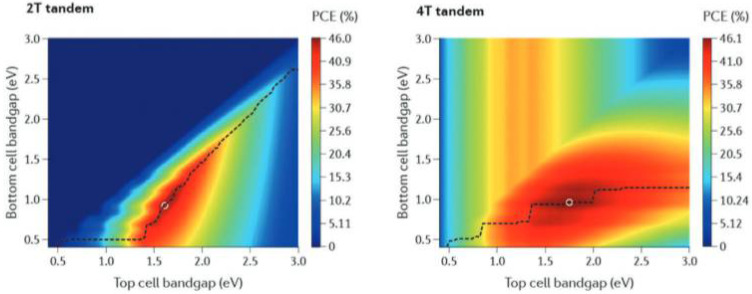
The schematic diagram of simulated optimized bandgap [28].

The perovskite materials play a role in absorbing incident light in the device. Hence, the photoelectric characteristics of the perovskite material play a key role in the photovoltaic characteristics of the solar cell. The perovskite materials with a wide bandgap and strong absorption in the visible and near-infrared regions are required to achieve efficient absorption. If the perovskite bandgap is too low, the open-circuit voltage (V_oc_) of the device is reduced accordingly despite the greater absorption of incident light. The bandgap can be adjusted by the elemental composition of perovskite materials [26]. For tandem solar cells, the bandgap width of the bottom and top cells must be adjusted to maximize the absorption of light and reduce the open-circuit voltage loss, thereby improving the power conversion efficiency [27]. The simulated maximum efficiency band adaptation is shown in Figure 3 [28]. For a 2T tandem cell, the top cell has a bandgap of 1.6 eV and the bottom cell has a bandgap of about 1.0 eV. For a 4T tandem cell, the bandgap of the top cell should be 1.7–1.8 eV and the bandgap of the bottom cell should be around 1.0 eV to achieve the highest efficiency.

**Figure 4:**
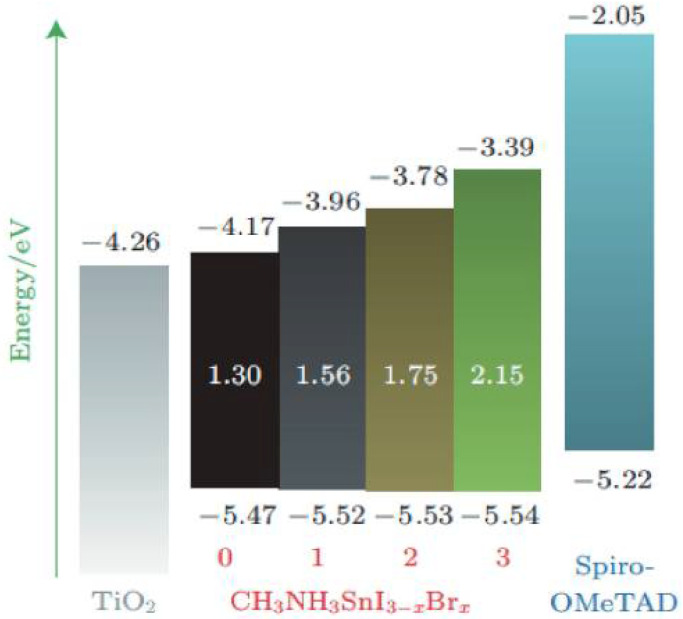
The relationship between Pb/Sn ratio and bandgap of perovskite [33].

Zhu et al. have prepared a narrow bandgap perovskite material by adjusting the ratio of Pb and Sn [29]. They have demonstrated that the surface properties are better than 50% when the Pb/Sn ratio is less than 50%. The relationship between Pb/Sn ratio and bandgap is shown in Figure 4. Dewi et al. have also fabricated a four-terminal silicon-based tandem perovskite solar cell by regulating the elemental composition of Br^−^ [30]. They have compared two different perovskite materials with bandgaps of 1.58 eV and 1.72 eV as top cells, and they studied the efficiency and effect of contrasting planar TiO_2_ and mesoporous SnO_2_ as electron transport layers. It has been concluded that the perovskite with a bandgap of 1.58 eV is more suitable as a top cell of the Si-based tandem cell, and the maximum PCE of the 4T tandem cell reached 25.5%. Table 1 shows the influence of PSC structure and operation on device performance.

Yao et al. have reported that a narrow bandgap solar cell, with a bandgap of 1.2 eV, could be fabricated by partially replacing Sn^4+^ with Sn^2+^ [31]. The PCE and short-circuit current of a single cell reached 19.58% and 29.81 mA cm^−2^, respectively. The conversion efficiency of a serially connected 4T tandem solar cell with a wide bandgap of 1.6 eV reached 23.26%. The et al. have replaced I-ions with Br-ions to improve the bandgap (1.73 eV) and solve the problem of photoinduced phase separation of wide-bandgap perovskite materials, which is suitable for constructing the top cell of tandem solar cells [32].

### 2.2. Doping-Induced Crystal Quality and Device Stability

The crystal structure of perovskite is easily damaged and decomposed under high-temperature or humid environments. The stability of perovskites is mainly related to thermal stability and water sensitivity, which mainly hinders the successful realization of PSCs. In general, the perovskite materials with MA or FA as cations exhibit strict requirements for ionic size. Hence, an extremely tiny lattice expansion or lattice distortion will greatly reduce the symmetry and structural stability of the perovskite material. During practical applications, the solar cells are required to work continuously at temperatures above 60 °C. Hence, the thermal stability of perovskite materials in a solar cell is critical for the long-term stability of solar cells. At the same time, the improvement in crystallinity of perovskite enhances the crystal quality and reduces the density of defect states, which are also crucial for solar cells [34].

Min et al. have proposed that the doping of methylenediammonium dichloride (MDACl_2_) in FAPbCl_3_ can stabilize the *α*-phase of perovskite layer [35]. It has been reported that the optimal concentration of MDACl_2_ is 3.8 mol%, resulting in a stable *α*-phase active layer and rendering little influence on the bandgap. The short-circuit current and PCE of the perovskite solar cell reached 26.1–26.7 mA cm^−2^ and 23.7%, respectively. After 600 h of full sunlight exposure, the power conversion efficiency still maintained 90% of the initial value. Wu et al. have proposed that a type of bilateral alkylamine (BAA) could be incorporated into perovskite to achieve passivation [36]. It has been demonstrated that the amino group in the bilateral alkylamine could form a coordination bond with Pb^2+^, thereby filling the gap between the A-sites of MAPbI_3_. Compared with the traditional amino molecule passivation, BAA can fill the gap with the hydrophilic amino side and the hydrophobic alkyl back to the outside, which could improve the moisture resistance of the perovskite layer. The p-i-n structure of ITO/PTAA/BAA-modified perovskite/C60/bathocuproine (BCP)/Cu solar cells exhibited excellent stability and a PCE of 21.5% under standard light intensity. It is noted that 90% of the initial efficiency has been maintained for 1000 h under dark conditions, and the PCE reached 90% after an initial 500 h of illumination.

Furthermore, organic and inorganic halide ions in hybrid perovskites exhibit low activation energy for migration (around 0.2–0.8 eV), facilitating the ionic migration through defects and escape via grain boundaries for a long range. Wei et al. have introduced rubrene in perovskite materials to attain cation–π interactions, which can effectively block the migration of organic cations and decrease the density of defects, strengthening the framework of perovskite crystal [37]. There is a champion device, with rubrene concentration of 10 mg mL^−1^, that achieved the optimal efficiency of 20.86%. In addition, the cation-immobilized perovskite films successfully inhibit the diffusion of iodine ions between different layers in PSCs during long-term operation. So, rubrene-based PSCs maintained 98% of the initial efficiency after 720 h under humidity (70%) at 25 °C. Wang et al. have introduced poly(ethylene oxide) (PEO) to stabilize MA^+^ and I^−^ by forming hydrogen interactions between PEO and the absorber material, restricting point defects and vacancies [38]. It has been demonstrated that PEO could improve the crystallization and stability of PSCs. Consequently, a device structure has been fabricated using ITO/NiO*_x_*/CH_3_NH_3_PbI_3_ (or PEO-CH_3_NH_3_PbI_3_)/PC_61_BM/A), rendering a PCE of >26%. Gao et al. utilized cesium chloride (CsCl) and lead bromide (PbBr_2_) to improve the stability of the black phase (*α*-phase) FAPbI_3_ and then fabricated a double cation (Cs, FA) perovskite solar cell, resulting in improved stability and photovoltaic performance [39]. The optimal amounts of CsCl and PbBr_2_ result in high crystallization and large grain size, enhancing the carrier lifetime. Furthermore, small crystals (PbI_2_) were found around the grain boundaries of the perovskite, stabilizing the crystallinity and passivating the defects of perovskite. It is a sensible method to modify the composition and structure of perovskite by doping. Thus, the crystallization and related properties of perovskite can be improved.

## 3. Transport Layers

In general, the configuration of perovskite solar cells is a sandwich structure, which includes an active layer that absorbs light, a *p*-type hole transport layer and an *n*-type electron transport layer [40]. As a large number of carriers originate from the active layer, the photocurrent of the solar cell is greatly reduced if these carriers are not efficiently transported. Therefore, it is necessary to utilize the electron transport layer and hole transport layer to timely separate the photo-generated carriers in the active layer [41]. Hence, the material and structure properties of the electron transport layer and hole transport layer influence the construction of high-performance perovskite solar cells.

An excellent electron/hole transport layer possesses good light transmittance, selective carrier separation and stability under light, heat and humidity. At present, the widely used transmission layer structures are mainly planar and mesoporous. The commonly used electron transport layer materials are mainly copolymers and metal oxides, such as PCBM, BCP, ZnO and TiO_2_. The hole transport layer materials are mostly composed of metal oxides and polymers, including Spiro-OMTED, PTAC, PTAA and NiO*_x_* [42].

### 3.1. Design of Electron Transport Layer

Chen et al. have synthesized nanocrystalline WO*_x_* as an electron transport layer using an ultra-low temperature (50 °C) solution-based process, which effectively avoided several problems, such as the high defect density of WO*_x_*. The power conversion efficiency of the resulting perovskite solar cell reached 20.77% [43].

**Figure 5:**
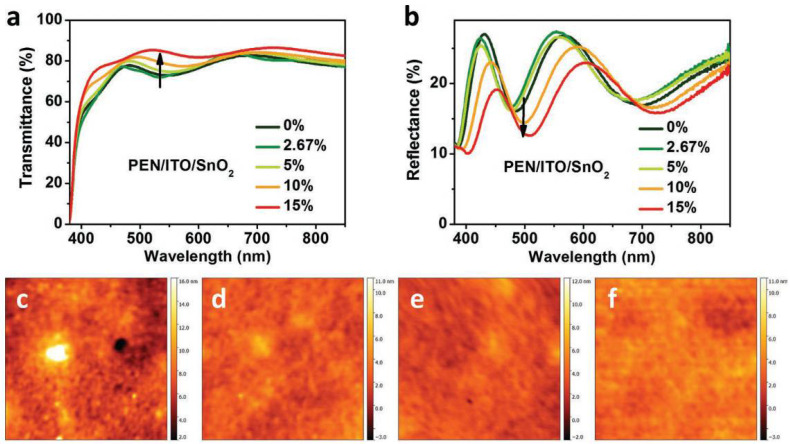
The influence of SnO_2_ concentration on surface performance, transmission and reflection of solar cells. (**a**,**b**) Transmission spectra and reflectance spectra of the structure of PEN/ITO/SnO_2_ with difference SnO_2_ concentration; AFM pictures of SnO_2_ film with different SnO_2_ concentration: (**c**) 2.67%, (**d**) 5%, (**e**) 10%, (**f**) 15% [44].

Huang et al. have utilized SnO_2_ as an electron transport layer in flexible PSCs and studied the influence of ETL thickness by adjusting the concentration of SnO_2_ [44]. It has been concluded that the optimal ratio of SnO_2_ is around 5%. The results revealed that the SnO_2_ improved the surface properties between the perovskite layer and electron transport layer, reduced the density of defect states and enhanced the absorption capacity perovskite layer. The AFM images of as-synthesized SnO_2_ on a flexible substrate and the corresponding transmission and reflection spectra are shown in Figure 5. The flexible solar cell, with the structure of polyethylene 2,6-naphthalate(PEN)/ITO/SnO_2_/perovskite/Spiro-OMeTAD/Ag, could finally achieve a PCE of 19.51% and maintain 90% of the initial PCE after 6000 cycles.

Jiang et al. have found that the incorporation of F8BT (poly(9,9-dioctylfluorene-co-benzothiadiazole)) into PCBM could solve the long-standing problems of poor film formation and inferior electronic mobility of PCBM [45]. F8BT is an optoelectronic polymer with excellent film-forming properties and exhibits better electron mobility than hole mobility. Therefore, F8BT can improve the performance of PCBM. Jiang et al. have reported that the addition of only 5 wt % F8BT could decrease the roughness of both PCBM and active layers [45]. Consequently, a solar cell, with a structure of Ag/PCBM/BCP/perovskite/NiO*_x_*/ITO, has been fabricated and demonstrated that the optimal amount of F8BT could significantly increase the IPCE, PCE, carrier concentration and EQE. The electrical impedance spectroscopy revealed that the addition of F8BT improved the device’s ability to separate electrons and holes and reduced the dark current. Li et al. have pioneered the production of uniform and compact TiO_2_ layers using the gas pumping method, which is usually called gas-phase quenching [46]. This thin, compact, pinhole-free TiO_2_ can also improve the crystalized quality of perovskite material and enhance the power conversion efficiency. Compared with conventional drying, the PSCs with gas-phase quenching exhibit high PCE (>20%), originating from the high short-circuit current density (J_SC_). Moreover, the as-fabricated device rendered high stability and retained 66% of the initial PCE after 550 h. On the other hand, the PCE of the device, which has naturally dried TiO_2_, is rapidly decreased in only 200 h under a humid environment (RT 40−60%). Meanwhile, this device with gas pumping TiO_2_ exhibits great potential for large-area applications. Yang et al. exploit a nanopatterned mesoporous TiO_2_ as ETL by the nanoimprinting method for nanopatterning [47]. They found that the PSC with 127 nm mesoporous TiO_2_ film could reach the highest PCE of about 15.83%. Cao et al. utilized the TaCl_5_ doped SnO_2_ as ETL in the n-i-p structure PSC [48]. After adopting TaCl_5_ doped SnO_2_ ETL, the open circuit voltage is increased from 0.97 to 1.08 V, and the PCE is increased from 16.38% to 18.23%.

### 3.2. Design of Hole Transport Layer

Until now, a large number of studies about different organic hole transport materials, such as Spiro-OMeTAD, PTAA and PEDOT:PSS, have been carried out to improve the efficiency and charge extraction. However, the development of various hole transport materials is expensive and leads to instability. On the other hand, a series of inorganic KTL materials, such as NiO, CuO, CuI, VO*_x_*, CuCrO_2_, CuGaO_2_, MoO*_x_* (as an interlayer), and WO*_x_*, are also considered due to their low cost, abundance and non-toxicity [49,50,51].

For small-molecule polymers, polyaniline (PANI) is an attractive photovoltaic material because of its excellent performance and low cost. Recently, Bourdo et al. have proposed that PANI could be doped with lignin sulfonic acid and camphor sulfonic acid to tune the work function and processed with DMSO to obtain LS-PANI-CSA. The introduction of LS-PANI-CSA as an HTL of an inverted perovskite solar cell decreases the interfacial roughness between the perovskite layer and HTL. At the same time, this structure can enhance the adhesion and dispersion of perovskite. Meanwhile, the interstitial spaces of the perovskite become smaller, and the obvious pinholes are not observed. After the experiment, the research team found that the utilization of LS-PANI-CSA HTL, with a thickness of 15 nm, could achieve optimal performance [52].

**Figure 6:**
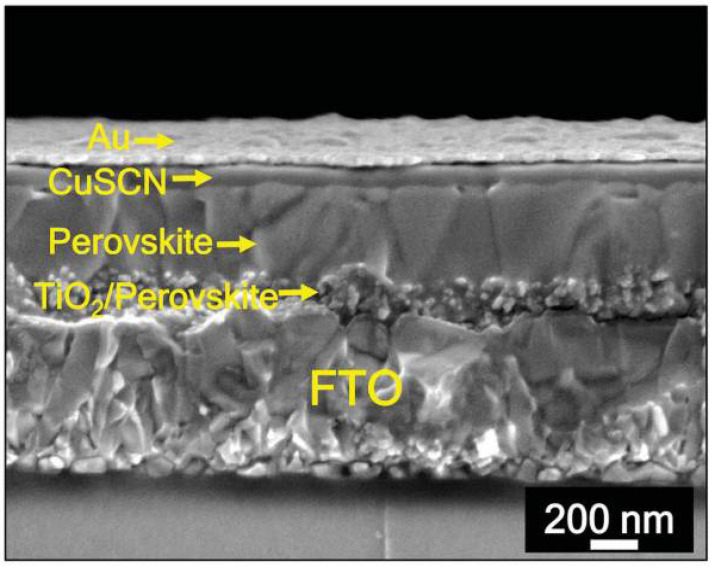
SEM images of the PSC with CuSCN and CuSCN/rGO as HTLs [54].

Moreover, carbon quantum dots, as a newly emerging optoelectronic material, are also being widely studied. Kim’s group has indicated that the combination of carbon QDs with NiO*_x_* could effectively reduce the J-V hysteresis loop, enhancing the stability of PSCs [53]. At the same time, this structure can improve the carrier transmission capacity and increase the short-circuit current and fill factor (FF). The PCE of the PSC with QDs/NiO*_x_* as an HTL was found to be 17.02%, which is significantly higher than the PSC with NiO*_x_* as an HTL (15.66%). Moreover, the PSC with QDs/NiO*_x_* as an HTL was left in the air for 190 h and retained 70% of the initial PCE. Milic et al. have demonstrated that CuSCN could be used instead of traditional Spiro-OMTED as a perovskite HTL due to its excellent hole transport capacity, thermal stability, solution-based processing and cost-effectiveness [54]. After a series of experiments, it has been reported that the PCE of PSC with CuSCN exceeded 20% and the device demonstrated excellent thermal stability. After covering the CuSCN with another layer of reduced graphene oxide (rGO), the operation stability of the device has also been greatly improved, and it reached 95% of the initial PCE after 1000 h operation at 60 °C. The structure of the device is presented in Figure 6. 

Furthermore, Li et al. have successfully synthesized a well-dispersed NiO*_x_* nanometer micelle solution with oleic acid (OA) to modify the dispersion of NiO*_x_* [55]. This nanometer micelle solution was introduced into the Spiro-OMTAD solution to fabricate a NiO*_x_*/Spiro hole transport bilayer. They have demonstrated that the hole transport bilayer possesses a higher hole transport rate and better stability than the pure Spiro because NiO*_x_* played an important role in improving the passivation and surface morphology of the perovskite layer. As a result, the device with a hole transport bilayer exhibited better performance than the Spiro-based PSC. Bogdanowicz et al. have used the4,4′-((1E,1′E)-((1,2,4-thiadiazole-3,5-diyl)bis(azaneylylidene))bis(methaneylylidene))bis(*N*,*N*-di-p-tolylaniline) (bTAThDaz) as the HTM. bTAThDaz can tune the HOMO-LOMO energy level to optimize the performance of perovskite solar cells. The PSC conversion efficiency of their fabricated structure of FTO/TiO_2_/perovskite/bTAThDaz/Ag reached 14.4% [56]. In summary, device efficiency can be improved by using different electron/hole transport materials or by modifying the transport layer.

## 4. Configurations of Perovskite Solar Cells

The structure of the PSC device is usually compared with the traditional dye-sensitized solar cells, which utilize an iodine-containing electrolyte as an HTL [57]. However, the device structure is close to the solid-state dye-sensitized solar cells because PSCs contain solid-state hole transport materials. To date, a variety of device structures have been proposed for perovskite solar cells [58]. The main structures of a single-cell PSC are mesoporous structure and planar structure. The main feature of the mesoporous structure is the introduction of semiconducting or insulating nanoparticles to assist electronic transport or perovskite film formation [59]. In the planar structure, the perovskite film is directly prepared without the introduction of a mesoporous skeleton, thereby making the perovskite preparation method more diverse and relaxing the constraints of fabrication conditions. Moreover, there are special device structures, such as hole-free transport structures, to simplify the process and reduce the production cost [60].

The basic structure of the tandem PSCs is briefly introduced in the first part (Figure 2) and it is mainly divided into two types: two-terminal tandem cells and four-terminal tandem cells. The manufacturing process of the four-terminal tandem cells is simple and does not require an additional active layer, but the two sub-cells are connected separately [21,61,62,63]. Hence, the utilization of four electrodes is easy to cause leakage current loss, and the manufacturing process becomes more complicated. For two-terminal tandem cells, the intermediate layer must have a certain degree of transparency and good electrical conductivity. This results in high requirements on the roughness, deposition temperature and solvent even though only two electrodes are involved in the intermediate layer [64]. It is worth noting that the selection of perovskite material for top and bottom cells is based on a bandgap.

### 4.1. Tandem Perovskite Solar Cells

**Figure 7:**
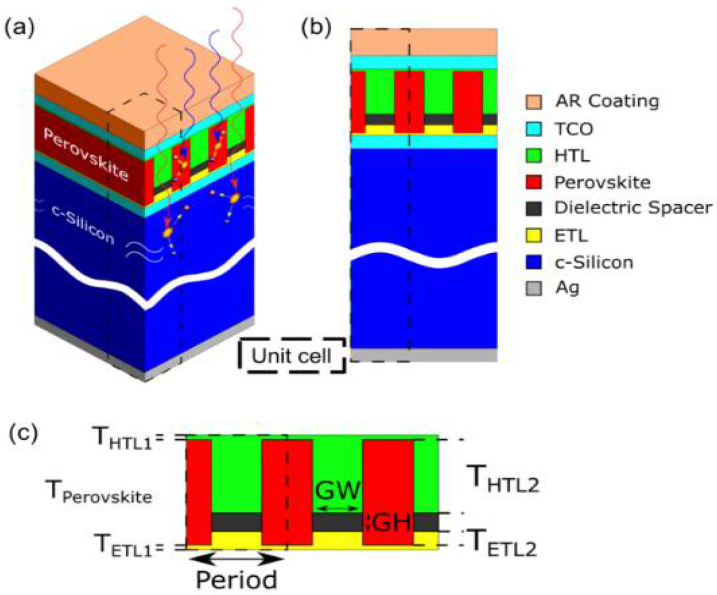
(**a**,**b**) The schematic illustration of tandem PSCs, (**c**) Details of the nanostructure [68].

Garcia-Camara’s group has incorporated a nanostructured layer, called a super-surface layer, into the perovskite layer, as shown in Figure 7, to introduce long-wavelength photons into the silicon bottom cell layer and disperse short-wavelength photons in the perovskite layer, resulting in improved absorption efficiency and power conversion efficiency of the PSCs. They have also introduced an antireflection layer to further reduce the loss of sunlight during the absorption process [65]. In addition to the incorporation of LiF or MgF antireflection layers on the surface of top cell, there are several ways to increase the transmitted light. For example, Hossain et al. have utilized a self-textured ZnO as an electron transport layer, which could also act as an antireflection coating and greatly reduce the processing cost [66]. Qiu et al. have directly used hydrogenated heterojunction silicon nanocrystals to embed an antireflection layer between perovskite and Si layers, increasing the light transmission and reducing the reflection. The final short-circuit current density reached 0.82 mA cm^−2^ [67].

Sahli et al. have directly constructed a fully textured perovskite-Si two-terminal tandem solar cell, which owns a high short-circuit current by increasing the sunlight absorption, reducing the absorption loss [69]. This structure differs from the conventional textured perovskite-Si solar cells because its structure is completely pyramid-shaped. First, both sides of Si were etched using KOH and, then, PECVD was employed to evaporate Si nanocrystals as an intermediate transport layer instead of ITO. This effectively reduced the deposition of the Spiro layer on the texture and reduced the leakage current caused by ITO deposition. Subsequently, a Spiro layer was evaporated, and a perovskite layer was formed using the spin-coating method. Then, an SnO_2_ layer and an IZO layer were formed using atomic layer deposition (ALD) and sputtering, respectively. Cai et al. have placed dielectric nanovertebras on the top of the solar cell and deposited silver nanoparticles on the bottom of the solar cell to increase the light absorption. The simulation results revealed that such a structure increases light absorption by 12.5% [70]. Manzoor et al. have also utilized textured structures to increase light absorption and employed the polydimethylsiloxane (PDMS) polymer to attain the desired texture; the PDMS polymer was placed on the surface of a silicon heterojunction to enhance light absorption [71].

**Figure 8:**
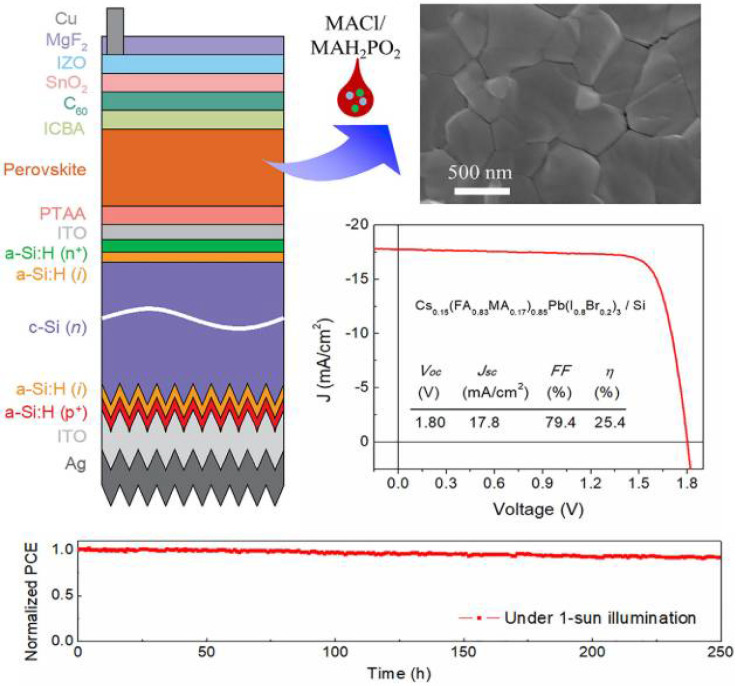
The structure and performance of the two-terminal tandem solar cell [72].

Chen et al. have improved the crystal quality of the wide-gap perovskite material by adding MACl and MAH_2_PO_2_ into Cs_0.15_(FA_0.83_MA_0.17_)_0.85_Pb (I_0.7_Br_0.3_)_3_ (E_g_ = 1.64 eV) [72]. These additives can increase the lattice constant and effectively reduce the density of defect states of perovskite. In the resulting device, the main role of MACl was to increase the crystalline size, and the main role of MAH_2_PO_2_ was to reduce defects and increase the stability of MACl after annealing. After doping, the power conversion efficiency of a single-cell solar cell reached 19.3%. In addition, the PCE of the two-terminal tandem solar cell which has the textured silicon bottom cell was as high as 25.4%. At the same time, the tandem cell still exhibited excellent stability. At room temperature and under standard sunlight, the PCE of the tandem solar cell is not reduced, even after 250 h. The structure and specific performance of the solar cell are shown in Figure 8. They have also utilized double-sided alkylamine groups to passivate MAPbI_3_, which greatly improved the thermal stability and humidity stability of the PSCs as well as increased the carrier lifetime from 278 to 889 ns [36].

Bush et al. have employed Cs^+^ cations to partially replace the FA^+^ cations and fabricated a Cs_0.17_FA_0.83_Pb(Br_0.17_I_0.83_)_3_-based single solar cell. Compared with MA-based solar cells, the stability has been greatly improved [73]. Under the relative humidity of 40% and at a temperature of 35 °C, the solar cell maintained a high PCE even after 1000 h. They have also fabricated a serially connected two-terminal tandem solar cell and demonstrated a PCE of 23.6%. Tong et al. have added GuaSCN into (FASnI_3_)_0.6_(MAPbI_3_)_0.4_ to significantly improve the surface flatness, crystallinity and spectral absorption of the Sn-Pb perovskite cells [74]. After the addition of 7 wt % GuaSCN, the optimal performance of Sn-Pb perovskite cells has been achieved with a minimum density of defect states. Moreover, the PCE reached 20.5%, and the short-circuit current was as high as 30.5 mA cm^−2^. The power conversion efficiency of the four-port tandem solar cell, where the GuaSCN-based cell was serially connected with a bottom cell (1.25 eV), reached 23.4%. Min et al. have utilized an FA and MA mixture to prepare a double-cation hybrid perovskite (FAPbI_3_)_0.95_(MAPbI_3_)_0.05_ solar cell [35]. One should note that a small amount of FAPbI_3_ was used to stabilize the structure and increase perovskite stability, rendering a power conversion efficiency of 23.73%. Overall, the fabrication of tandem cells is a hopeful way to break through the S-Q limit.

### 4.2. HTL/ETL-Free Perovskite Solar Cells

To ensure the effective separation of excitons, whether it is an n-i-p forward perovskite solar cell or a p-i-n reverse perovskite solar cell, there are complete electron transport layers and hole transport layers on both sides of the perovskite. Although the current cost of perovskite materials is relatively low and the cost of device fabrication and ETL can be controlled, the cost of hole transport layers, such as Spiro-OMeTAD, greatly increases the cost of PSCs. As perovskite possesses hole transport properties, Etgar et al. have employed perovskite as HTL and a light-absorbing material. The power conversion efficiency of the resulting device was found to be 5.5%, which proved the feasibility of HTL-free PSCs [75]. Moreover, the device performance was further improved by optimizing the surface morphology, and the PCE exceeded 10% [76].

**Figure 9:**
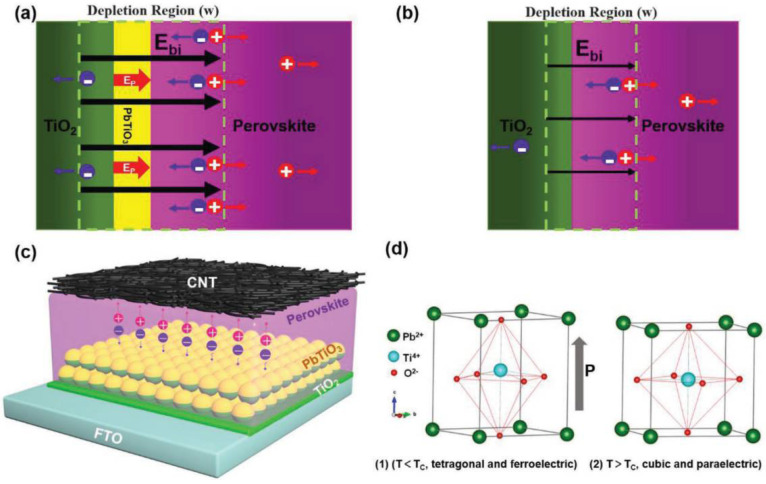
The schematic illustration of the ferroelectric and HTL-free PSCs, (**a**,**b**) The depletion region in PSCs with and without the PbTiO_3_ interlayer, (**c**) Schematic structures of the ferroelectric layer-based PSC device, (**d**) Lattice structures of PbTiO_3_ [78].

Ding et al. have used an rGO-doped carbon electrode to replace the traditional hole transport layer and fabricate HTL-free perovskite solar cells [77]. They have incorporated a certain amount of NiO into rGO to match the energy level between perovskite and carbon electrode, thereby improving the power conversion efficiency of solar cells. Yang et al. reported that the PbTiO_3_ ferroelectric layer could be inserted between the electron transport layer and perovskite layer to fabricate HTL-free PSCs [78]. The ferroelectric layer generates a polarization field between the electron transport layer and perovskite layer. The direction of the polarization field is consistent with the direction of the built-in electric field, which effectively promotes the separation of electrons and holes. The PCE of the corresponding HTL-free PSC reached 16.37%, and the operating principle is schematically illustrated in Figure 9.

Huang et al. have introduced a thin polar non-conjugated small-molecule electrolyte layer to modify the ITO layer and obtain ETL-free PSCs with a low hysteresis loop [79]. The role of the small-molecule electrolyte layer as an additional layer to adjust the work function (WF) of ITO has been confirmed. Overall, the photovoltaic properties, such as PCE, hysteresis, J_sc_, V_oc_ and FF, are enhanced due to the better alignment of energy levels. As a result, the PCE of the modified device reached 20.55%, which is much higher than the ITO-based ETL-free device (12.81%) and comparable to the PSCs with a conventional structure. Fan et al. have in situ prepared and incorporated AgI QDs into the perovskite material to fabricate an HTL-free PSC with high performance [80]. They have demonstrated that an MAPbI_3_:AgI (QDs) cross-blended structure deprived of AgI QDs and perovskite could be observed. There are several positive effects of AgI QDs, including better crystallization, fewer defects, and excellent distribution of Fermi level/work function (WF) between the perovskite layer and ITO layer.

Similarly, Cheng et al. have designed, synthesized and utilized hydroxylethyl-functionalized imidazolium iodide ionic liquid to modify FTO [81]. It has been reported that the ionic liquid could successfully decrease the surface potential and WF of FTO, thereby efficiently blocking the diffusion of holes and optimizing the alignment of energy levels. Consequently, the PCE of corresponding PSC increased from 9.01% to 17.31%, and the stability and hysteresis were also improved. HTL/ETL-free PSCs are a novel and cost-effective structure that deserves further research.

### 4.3. Perovskite Solar Cells and Graphene

Graphene is an excellently honeycomb-structured material, which has garnered much attention in optoelectronic devices, such as OPVs, LEDs, OLEDs and photodetectors, because of its unique physical, chemical and mechanical properties as well as extraordinary thermal, electronic and hydrophobic properties [82]. Moreover, graphene oxide derivatives can be processed in a solution state [83]. In PSCs, graphene is mainly used as a barrier layer to impede the degradation of the perovskite layer and HTL due to its high hole transport velocity and low resistance [84]. Hence, the utilization of graphene in PSCs has also been widely studied.

**Figure 10:**
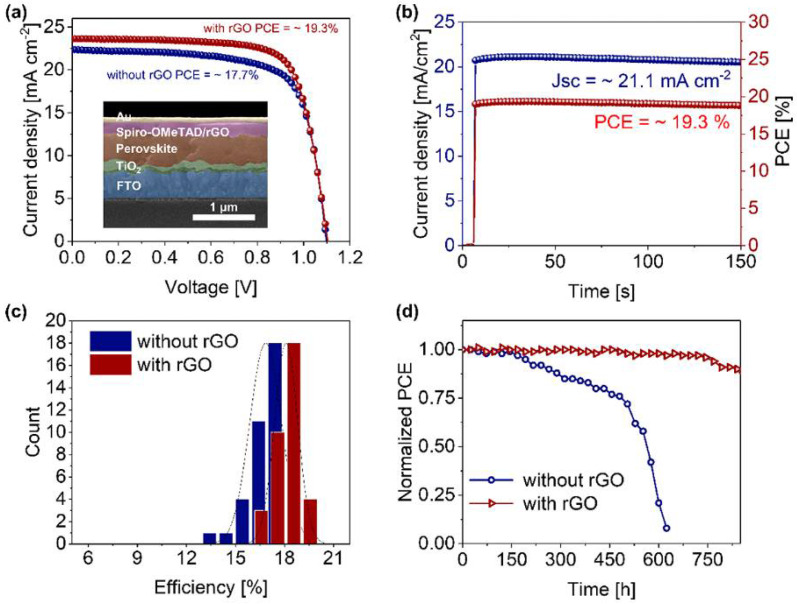
(**a**) J-V curve of PSC device with/without rGO, (**b**) Steady curve of champion device, (**c**) PCE distribution map, (**d**) Stability testing of PSCs [86].

Lee et al. have utilized an improved method to transfer the graphene layer instead of conventional copper-based etching [85]. The CVD-synthesized graphene can form better contact with the perovskite layer. They have introduced three layers of impermeable graphene with atomic thickness as an interfacial barrier to obstruct moisture, I-ions and Au diffusion. Owing to the high hole mobility, the PSC device with Au, CuSCN, graphene (3 layers), a perovskite layer, PTAA and FTO exhibited a negligible change in PCE (15.2 to 15.8%) and a significant increase in moisture resistance. The graphene layer significantly reduced the moisture intrusion, and the corresponding device maintained > 94% of the initial PCE under 50% RH for 30 days. Similarly, Guo et al. have employed rGO as an additive in Spiro-OMeTAD HTL of PSCs to impede the diffusion of Li^+^ from Li-TFSI [86]. They have indicated that the Li^+^ ions bring high hygroscopicity, which can exacerbate the degradation of the Spiro-OMeTAD HTL layer and perovskite layer. Therefore, rGO can effectively improve the stability of PSC and PCE due to its hydrophobic behavior and excellent conductivity. As shown in Figure 10, the presence of rGO in the Spiro-OMeTAD layer effectively obstructs the diffusion of Li^+^ ions, thereby improving the efficiency and stability of this device.

Sahin et al. have utilized two different amine sources, i.e., dihexylamine (DHA) and 2-ethylhexylamine (2EHA), to modify graphene oxide (GO) [87]. Then, GO and modified GOs (mGOs) were inserted between the halide mesoporous perovskite layer and hole transport layer as a buffer layer. The device with mGO exhibited much better surface morphology and photovoltaic performance than the GO-based device. The increase in electronic transport of the samples with DHA-GO and 2EHA-GO buffer layers can be attributed to the full coverage of the layer and high hole transport velocity. Mann et al. have combined sulfonic-oxidized graphene with PEDOT:PSS to form PEDOT:PSS/SrGO because SrGO possesses excellent water and oxygen resistance, a desirable band structure and high carrier transport capacity [88]. When PEDOT:PSS/SrGO was used as an HTL in PSCs, it improved the stability of perovskite solar cells and enhanced the carrier separation and transmission capability to a certain extent. The results revealed that the utilization of PEDOT:PSS/SrGO significantly increased the PCE from 13.5% to 16.01% and enhanced the stability of PSC. Hence, the PSC maintained 85% of the initial PCE after being left in the air for 30 days, demonstrating that SrGO greatly improved the water and oxygen resistance. The combination of rGO with metal oxides is a feasible method to improve charge extraction and device stability. Hong et al. have employed nitrogen-doped graphene oxide (NGO) to control oxygen vacancies in SnO_2_ and improve the efficiency of photovoltaic devices [89]. The study confirmed the passivation of oxygen vacancy defects, where Sn^2+^ is turned into Sn^4+^ due to the presence of NGO. The resulting device rendered a PCE of 16.5% with negligible hysteresis and significantly improved V_oc_ and FF. As an excellent conductive material and water barrier material, graphene can effectively improve the performance and stability of PSCs.

**Table 1:** Influence of PSC structure and operation on device performance.

Structure	Operation	J_sc_ (mA/cm^2^)	V_oc_ (V)	FF (%)	PCE (%)	Ref.
4T tandem	Adjust Pb/Sn ratio	-	-	-	25.5	[30]
4T tandem	Adjust Pb/Sn ratio	-	-	-	23.3	[31]
FAPbCl_3_	MDACl_2_ doping	-	-	-	23.7	[35]
MAPbI_3_-DAP	BAA doping	22.5	1.18	81.7	21.7	[36]
Single cell	Cation-immobilized perovskite films	24.2	1.11	77.7	20.8	[37]
MAPbI_3_	PEO doping	22.4	1.10	77.8	19.2	[38]
MAPbI_3_	WO*_x_* as ETL	24.8	1.06	79.1	20.8	[43]
Single cell	Optimize SnO_2_	23.1	1.11	76.1	19.5	[44]
MAPbI_3_	Optimize PCBM	19.3	1.05	74.7	15.3	[45]
MAPbI_3_	In situ solidification of the TiO_2_	24.0	1.06	80.3	20.4	[46]
MAPbI_3_	Nanopatterned mp-TiO_2_	24.6	0.89	72.0	15.8	[47]
MAPbI_3_	LS-PANI-CSA as HTL	19.5	0.82	68.0	10.8	[52]
MAPbI_3_	Carbon quantum dot-incorporated nickel oxide	19.6	1.08	77.5	16.4	[53]
(FAPbI_3_)*_x_*(MAPbBr_3_)_1−*x*_	NiO*_x_*/Spiro hole transport bilayers	23.8	1.14	79.8	21.7	[55]
MAPbI_3_	bTAThDaz as HTL	23.2	0.93	0.6	14.4	[56]
4T tandem	Hybrid metasurface and textured back contact	-	-	-	20.5	[65]
2T tandem	Fully textured Structure	19.5	1.79	73.1	25.5	[69]
2T tandem	PDMS scattering layer	20.2	1.10	75	16.7	[71]
2T tandem	MACl and MAH_2_PO_2_	17.8	1.80	79.4	25.4	[72]
2T tandem	Infrared-tuned silicon heterojunction bottom	18.1	1.65	79.0	23.6	[73]
4T tandem	GuaSCN doping	-	-	-	25.4	[74]
MAPbI_3_	P-type doping of rGO/NiO	19.3	0.84	52	8.5	[77]
HTL-free	Ultrathin ferroelectric perovskite oxide layer	23.5	0.93	75	16.4	[78]
ETL-free	PNSME layer energy band alignment optimization	23.6	1.15	75.7	20.5	[79]
HTL-free	AgI Quantum Dots	22.9	1.01	70.8	16.4	[80]
CsFAMAPbI_3−*x*_Br*_x_*	Graphene interfacial diffusion barrier	21.3	1.01	70.9	15.2	[85]
MAPbI*_x_*Br_3−*x*_	HTL modifying by adding reduced graphene oxide (rGO)	23.6	1.10	74.2	19.3	[86]
CH_3_NH_3_PbI_3__−*x*_Cl*_x_*	Amine-modified graphene oxide buffer laye	24.3	0.93	58	13.5	[87]
MAPbI_3_	PEDOT:PSS/SrGO hole interfacial laye	19.4	1.04	80.5	16.1	[88]
Rb_0.05_(FA_0.83_MA_0.17_) _0.95_Pb(I_0.83_Br_0.17_)_3_ + CsI	Graphene-oxide-treated tin oxide layer	18.8	1.17	74.9	16.5	[89]

## 5. Conclusions

In conclusion, the current review primarily describes the recent research progress on PSCs and highlights various strategies to improve the stability and efficiency of PSCs. The impact of additive engineering on band alignment and passivation has been discussed in detail. One should note that the introduction of different additives, including halogen elements and organic cations, is an effective route to optimize the band alignment, which can also enhance the stability and crystallization quality. Moreover, from the viewpoint of PSC configuration, the hole and electron transport layers play an important role in carrier excavation and device stability. Different organic and inorganic materials, which are commonly used for HTL and ETL, are also discussed from the viewpoints of material properties and device performance. In addition, the recent studies on different types of tandem PSCs and HTL/ETL-free PSCs are also summarized. Lastly, the successful incorporation of widely used graphene in PSCs is discussed as an effective strategy to enhance PCE and stability. For the successful realization of PSCs, several critical requirements are summarized and listed: (1) light absorption should be further increased without compromising the light loss, (2) voltage deficit and shunt of the PSC device should be minimized, (3) the energy bands of adjacent layers should be aligned to decrease the energy offset, and (4) the stability of perovskite structure in the operational environment should be further improved. In comparison with Si-based solar cells, PSCs are promising for the successful realization of solar technology due to their continuously increasing PCE; however, the stability of the perovskite structure should be addressed from fundamental science and applications perspectives. For the further development of PSCs and subsequent commercial applications, studies on stability, large size, and stacked structures should be carried out.

## Data Availability

Not applicable.
